# Convergence of miRNA Expression Profiling, α-Synuclein Interacton and GWAS in Parkinson's Disease

**DOI:** 10.1371/journal.pone.0025443

**Published:** 2011-10-07

**Authors:** Madalena Martins, Alexandra Rosa, Leonor C. Guedes, Benedita V. Fonseca, Kristina Gotovac, Sara Violante, Tiago Mestre, Miguel Coelho, Mário M. Rosa, Eden R. Martin, Jeffery M. Vance, Tiago F. Outeiro, Liyong Wang, Fran Borovecki, Joaquim J. Ferreira, Sofia A. Oliveira

**Affiliations:** 1 Neurological Clinical Research Unit, Instituto de Medicina Molecular, Lisboa, Portugal; 2 Instituto Gulbenkian de Ciência, Oeiras, Portugal; 3 Unidade Ciências Médicas, Centro Competências das Ciências da Vida, Universidade da Madeira, Funchal, Portugal; 4 Serviço de Neurologia, Hospital de Santa Maria, Centro Hospitalar Lisboa Norte, Lisboa, Portugal; 5 Department for Functional Genomics, Center for Translational and Clinical Research, University of Zagreb School of Medicine, University Hospital Center Zagreb, Zagreb, Croatia; 6 Hussman Institute for Human Genomics, Miller School of Medicine, University of Miami, Miami, Florida, United States of América; 7 Cell and Molecular Neuroscience Unit, Instituto de Medicina Molecular, Lisboa, Portugal; 8 Instituto de Fisiologia, Faculdade de Medicina, Universidade de Lisboa, Lisboa, Portugal; 9 Department of Neurodegeneration and Restaurative Research, Center for Molecular Physiology of the Brain, University Medizin Gottingen, Gottingen, Germany; Telethon Institute of Genetics and Medicine, Italy

## Abstract

miRNAs were recently implicated in the pathogenesis of numerous diseases, including neurological disorders such as Parkinson's disease (PD). miRNAs are abundant in the nervous system, essential for efficient brain function and play important roles in neuronal patterning and cell specification. To further investigate their involvement in the etiology of PD, we conducted miRNA expression profiling in peripheral blood mononuclear cells (PBMCs) of 19 patients and 13 controls using microarrays. We found 18 miRNAs differentially expressed, and pathway analysis of 662 predicted target genes of 11 of these miRNAs revealed an over-representation in pathways previously linked to PD as well as novel pathways. To narrow down the genes for further investigations, we undertook a parallel approach using chromatin immunoprecipitation-sequencing (ChIP-seq) analysis to uncover genome-wide interactions of α-synuclein, a molecule with a central role in both monogenic and idiopathic PD. Convergence of ChIP-seq and miRNomics data highlighted the glycosphingolipid biosynthesis and the ubiquitin proteasome system as key players in PD. We then tested the association of target genes belonging to these pathways with PD risk, and identified nine SNPs in *USP37* consistently associated with PD susceptibility in three genome-wide association studies (GWAS) datasets (0.46≤OR≤0.63) and highly significant in the meta-dataset (3.36×10^−4^<p<1.94×10^−3^). A SNP in *ST8SIA4* was also highly associated with PD (p = 6.15×10^−3^) in the meta-dataset. These findings suggest that several miRNAs may act as regulators of both known and novel biological processes leading to idiopathic PD.

## Introduction

Parkinson's disease is a neurodegenerative disorder clinically characterized by bradykinesia, muscle rigidity, resting tremor and, in more advanced stages, postural instability. Its main pathological hallmark is the loss of over 70% of the dopaminergic neurons (DNs) within the *substantia nigra* (SN), leading to functional deficits in the basal ganglia circuitry due to reduced levels of dopamine. The identification of several genes causing monogenic forms of PD (e.g. *α-synuclein* [*SNCA*], *Parkin*, *DJ-1*, *PINK1*, *LRRK2*) has led to the generalized view that protein misfolding, mitochondrial dysfunction, impaired oxidative stress response, and altered function of the ubiquitin-proteasome system are central pathogenic mechanisms underlying the familial forms of PD [1]. However, far less is known about the molecular mechanisms underlying idiopathic PD.

miRNAs have recently emerged as an important class of small RNAs (∼22 nucleotides) that act as post-transcriptional regulators of gene expression by base-pairing with their target mRNAs. They regulate neuronal processes such as brain morphogenesis, neuronal cell fate and differentiation, and transcription of neuronal-specific genes [2]. Recent studies have linked several miRNAs to sporadic PD. miR-133b was found to be specifically enriched in midbrain DNs of normal individuals and reduced in PD patients [3]. *In vitro*, miR-133b was found to regulate DN maturation and function through a negative feedback loop with *Pitx3*, a transcription factor that activates midbrain DN gene expression [3]. miR-433 binds to a polymorphism in the promoter region of the *fibroblast growth factor 20* gene (*FGF20*) which is associated with PD [4]. The risk allele disrupts the binding site of miR-433, resulting in enhanced translation of *FGF20* and increased α-synuclein expression [4]. miR-7 and miR-153 were shown to be predominantly expressed in the brain and to regulate α-synuclein expression levels [5]–[6]. In addition, mutant *LRRK2* was found to negatively regulate translational repression mediated by miRNAs [7]. None of these studies reported genome-wide miRNA expression profiling in the blood of PD patients. Since previous studies were restricted to brain tissue and limited in the number of samples and miRNAs evaluated, we sought to investigate the role of miRNAs in idiopathic PD by performing microarray expression profiling in blood samples of PD patients and controls.

α-synuclein is a key player in PD. It is the main component of the protein aggregates accumulated in the brain of patients, a pathologic hallmark of this disease. Further support for the implication of this molecule in PD has been gathered from GWAS [8]–[13] where the genetic architecture of the most common forms of PD has been investigated. Genome-wide significance has been reached in three of these GWAS [11]–[13], and *SNCA* and the *MAPT* region have been confirmed as major PD risk loci. In addition, mutations (e.g. A53T, A30P, E46K, and gene duplications and triplications) and polymorphisms in *SNCA* are linked both to monogenic and complex forms of PD, respectively [14]. Despite its recognized importance, the molecular mechanisms underpinning its role in PD are not yet clear. Although pathways like the ubiquitin proteasome system and chaperone-mediated autophagy have been implicated in α-synuclein turnover, our knowledge of its biology is still limited [15]. Here, we used ChIP-seq as an unbiased approach to identify α-synuclein binding sites genome-wide. This technology captures the full spectrum of actions of this molecule across the genome, providing information that will not only provide insights into the putative roles of α-synuclein in the nucleus, but also connect it to previously unanticipated cellular processes.

miRNAs have been studied for their direct or indirect action on α-synuclein [4]–[6] but they are predicted to target a wide range of genes. Given the centrality of α-synuclein in PD, here we propose to go one step further and not only to look at its interactions with other molecules and pathways but also to look into the cross-talk between the α-synuclein interactome and miRNAs.

To identify novel genes and pathways associated with PD, we followed an integrative approach, intersecting the results from these complementary genome-wide analyses to further test them in a GWAS meta-dataset, gaining further insight into the molecular mechanisms involved. Importantly, a PD miRNA signature with 18 miRNAs was obtained and two pathways (the glycosphingolipid biosynthesis - ganglioseries and the protein ubiquitination pathway) were highlighted as key players in PD pathogenesis. Furthermore, 3 miRNAs emerged as main modulators of these two pathways (miR-30b, miR-30c and miR-26a), and two genes (*USP37* and *ST8SIA4*) were found significantly associated with PD susceptibility.

## Results

### miRNA expression profiling

The principal demographic and clinical characteristics of the nineteen unrelated idiopathic PD patients and thirteen controls included in the miRNA profiling study are summarized in Table 1. Recruited patients covered the full spectrum of early to advanced PD (Hoehn & Yahr stage 1 to 5) and age was comparable across the two groups (average age-at-examination ± SD of 65.1±4.4 years in patients and 64.4±5.9 years in controls).

**Table 1:** Principal demographic and clinical characteristics of the miRNA profiling study participants.

ID	Affection status	Age	Sex	AAO	Years since onset	Hoehn & Yahr stage	Schwab & England score	UPDRS III score	UPDRS I+II+III score
P1	PD	58	M	50	8	1	90%	6	12
P2	PD	60	M	57	2	2	100%	3	8
P3	PD	61	M	56	5	1.5	90%	10	15
P4	PD	62	M	55	7	2.5	70%	17	50
P5	PD	64	M	61	3	2.5	90%	20	28
P6	PD	65	M	63	2	2.5	90%	12	15
P7	PD	68	M	55	13	4	30%	41	58
P8	PD	70	M	50	20	2.5	80%	16	21
P9	PD	71	M	60	10	2.5	50%	52	87
P10	PD	71	M	61	11	2.5	80%	23	45
P11	PD	58	F	45	13	2.5	70%	38	63
P12	PD	61	F	50	11	2.5	60%	29	41
P13	PD	62	F	45	17	2.5	70%	35	70
P14	PD	64	F	60	4	2.5	90%	24	31
P15	PD	67	F	65	2	2.5	90%	20	26
P16	PD	68	F	60	8	2	80%	12	16
P17	PD	68	F	59	9	3	80%	24	29
P18	PD	69	F	56	13	2.5	40%	31	93
P19	PD	70	F	62	8	5	NA	71	106
C1	CTRL	63	M						
C2	CTRL	64	M						
C3	CTRL	66	M						
C4	CTRL	71	M						
C5	CTRL	73	M						
C6	CTRL	51	F						
C7	CTRL	57	F						
C8	CTRL	60	F						
C9	CTRL	63	F						
C10	CTRL	66	F						
C11	CTRL	66	F						
C12	CTRL	67	F						
C13	CTRL	70	F						

PD: Parkinson's Disease; CTRL: Control; M: Male; F: Female; AAO: Age-at-onset; NA: Not available; UPDRS III: Unified Parkinson's disease rating scale Part III; UPDRS I+II+III: Unified Parkinson's disease rating scale Part I, Part II and Part III.

We conducted miRNA expression profiling in PBMCs of the study participants using Exiqon-developed miRCURY™ LNA microarrays spotted with four replicate probes for 763 human miRNAs (733 from miRBase (http://www.mirbase.org/) database release 13 plus 30 miRPlus novel human miRNAs from Exiqon). We found that 490 of these miRNAs were expressed in our samples. Differentially expressed miRNAs were identified using a linear modelling approach for each miRNA to estimate the fold-changes and standard errors, followed by an empirical Bayes smoothing to moderate the standard errors [16]. Empirical Bayesian methods provide more stable results even when the number of arrays is small. Taking a B-statistic (logarithm of the posterior odds that a gene is differentially expressed) threshold of 1, eighteen miRNAs were found differentially expressed (Table 2). Interestingly, all of these miRNAs are under-expressed in PD samples (fold-changes ranging from −1.41 to −3.02). To globally validate the microarray results, we performed qRT-PCR for five miRNAs, three of which had negative fold-changes (miR-126*, miR-32, and miR-101), and two had positive fold-changes (miR-15b and miR-550) in PD cases. Only miR-126* was significantly under-expressed in our microarray experiment (B≥1), while the other four miRNAs tested had B-statistics below 1. qRT-PCR supported our microarray findings (Figure S1) in terms of directionality (positive versus negative fold-changes) and strength of significance of differential expression (the lowest p-value in the qRT-PCR experiment was obtained for miR-126* which was the only miRNA among those five with a B-statistic greater than 1).

**Table 2:** miRNAs differentially expressed in PBMCs of PD patients and controls.

miRNA ID	miRBase accession	Fold-change PD/CRTL	t-statistic^1^	p-value^2^	Adjusted p-value^3^	B^4^
miR-335	MIMAT0000765	−1.78	−5.26	8.78E-06	1.75E-03	3.53
miR-374a	MIMAT0000727	−1.97	−5.24	9.21E-06	1.75E-03	3.49
miR-199a-3p/miR-199b-3p	MIMAT0000232	−2.18	−5.04	1.66E-05	1.76E-03	2.95
miR-126*	MIMAT0000444	−3.02	−4.94	2.26E-05	1.76E-03	2.68
miR-151-3p	MIMAT0000757	−1.53	−4.93	2.33E-05	1.76E-03	2.65
miR-199a-5p	MIMAT0000231	−1.93	−4.87	2.79E-05	1.76E-03	2.48
miR-151-5p	MIMAT0004697	−1.65	−4.69	4.73E-05	2.09E-03	2.00
miR-126	MIMAT0000445	−1.98	−4.68	4.75E-05	2.09E-03	2.00
miR-29b	MIMAT0000100	−2.36	−4.67	4.95E-05	2.09E-03	1.96
miR-147	MIMAT0000251	−1.41	−4.58	6.35E-05	2.14E-03	1.74
miR-28-5p	MIMAT0000085	−1.59	−4.57	6.58E-05	2.14E-03	1.71
miR-30b	MIMAT0000420	−1.77	−4.56	6.78E-05	2.14E-03	1.68
miR-374b	MIMAT0004955	−1.53	−4.44	9.73E-05	2.53E-03	1.35
miR-19b	MIMAT0000074	−2.47	−4.41	1.04E-04	2.53E-03	1.29
miR-30c	MIMAT0000244	−1.62	−4.41	1.06E-04	2.53E-03	1.27
miR-29c	MIMAT0000681	−2.01	−4.40	1.07E-04	2.53E-03	1.26
miR-301a	MIMAT0000688	−2.47	−4.37	1.19E-04	2.65E-03	1.17
miR-26a	MIMAT0000082	−1.72	−4.34	1.30E-04	2.75E-03	1.08

PD: Parkinson's Disease; CTRL: Controls;

1 Moderated t-statistic;

2 Raw p-value;

3 Adjusted p-value using the Benjamini and Hochberg method [60] or q-value;

4 B-statistic or log odds that the gene is differentially expressed [16].

**Figure 1:**
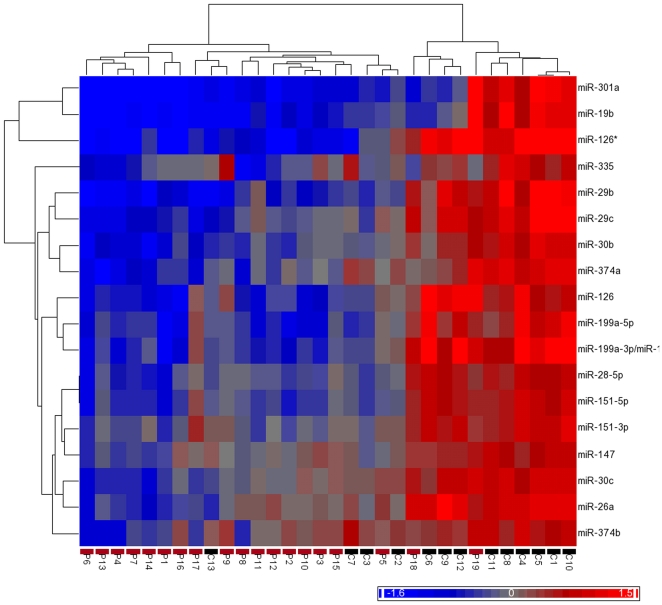
Unsupervised hierarchical clustering of the eighteen differentially expressed miRNAs (rows) for the 32 samples (columns). The sample ID and a heat-map indicating the disease state of each subject in a color code (black for controls and red for cases) are shown at the bottom of the plot. The color scale at the bottom right illustrates the relative expression level of a miRNA across all samples: red represents an expression level above mean, and blue represents expression lower than the mean. The clustering is performed on log_2_(sample/reference pool) ratios of the 18 differentially expressed miRNAs, using the Euclidean distance function and the average linkage clustering methods.

Unsupervised hierarchical clustering (HC) of the samples using the 18 differentially expressed miRNAs reveals that PD cases and controls cluster into separate groups with the exception of controls C2, C3, C7, and C13 and cases P18 and P19 (Figure 1). A similar pattern is observed using the principal component analysis (PCA) (Figure S2), where the first principal component explains 84.1% of the variance and allows a clear separation of most samples according to affection status. Supporting the consistency of our results, analysis of variance revealed that the main source of variation in the expression data is the individual's affection status (Figure S3), and we typically observed similar expression patterns for miRNAs derived from the same stem-loop precursor (e.g. co-clustering in Figure 1 of miR-199a-5p and miR-199a-3p, and of miR-151-5p and miR-151-3p, which derive from the miR-199a and miR-151 stem-loop precursors, respectively).

### Predicted target genes and pathway analysis

In an effort to place these miRNA expression profiling findings into a biological context and to generate new testable hypotheses, and given that miRNAs have very few experimentally validated targets, we first performed an *in silico* search for predicted target genes of the 18 differentially expressed miRNAs. In view of the fact that no particular target prediction software has consistently shown to be superior to all others, we used the “Predicted Targets” tool in the miRecords resource [17] which integrates the results of eleven established miRNA target prediction algorithms. Six softwares (miRanda v.1.9 [18], MirTarget2 v.2.0 [19], PicTar [20], PITA v.6 [21], RNAhybrid v.2.2 [22], and TargetScan v.4.1 [23]) generated almost all (>95%) of the predicted target genes for the miRNAs of interest. These six softwares rely on very distinct principles and methods, namely near-perfect base complementarity in the “seed” region, evolutionary interspecies conservation, miRNA-mRNA duplex thermodynamic stability, target site accessibility, and machine learning techniques. Given that these predictions have not been experimentally validated, we opted for a stringent threshold of at least six softwares to select the predicted miRNA target genes for follow-up. In this case, seven miRNAs (miR-374a, miR-199a-3p/miR-199b-3p, miR-126*, miR-151-5p, miR-126, miR-147, and miR-374b) had no predicted target genes, and the remaining eleven miRNAs were predicted to target 662 unique genes (Table S1).

To correlate expression findings with relevant biological processes and pathways, we performed pathway analysis using the manually-curated literature-based Ingenuity Pathway Analysis (IPA) database. Among the 662 unique predicted target genes used to query the database, 553 were eligible for functions/pathway/list analyses. The top ten canonical pathways over-represented among our target genes were (Figure S4A and Table S2): glycosphingolipid biosynthesis - neolactoseries (5 molecules), semaphorin signaling in neurons (7 molecules), DNA methylation and transcriptional repression signaling (4 molecules), retinoic acid receptor (RAR) activation (13 molecules), hypoxia signaling in the cardiovascular system (8 molecules), glycosphingolipid biosynthesis - ganglioseries (4 molecules), O-glycan biosynthesis (4 molecules), protein ubiquination pathway (16 molecules), synaptic long-term potentiation (9 molecules), and nicotinate and nicotinamide metabolism (8 molecules).

### α-synuclein target genes

High-throughput microarray studies typically generate too many leads to follow-up, requiring subsequent validation steps. To narrow down the genes for further investigation in association studies, we intersected the results from the miRNA pathway analysis with those from a study aimed at unveiling genomic targets for α-synuclein modulation (interacton), which may suggest novel unanticipated pathogenic pathways in PD.

To identify α-synuclein binding sites throughout the genome, we performed chromatin immunoprecipitation (ChIP) with an anti-α-synuclein antibody in cultured human H4 neuroglioma cells expressing endogenous levels of the protein. Next, high-throughput sequencing of the co-immunoprecipitated genomic DNA fragments was performed. Mapping the sequenced fragments to the reference genome gives a profile of the DNA regions that are enriched (“peaks”) in the pulldown, with a peak being defined as a region that contains significantly more reads from an experimental pulldown than from a negative control (ChIP sample and total input, respectively). The analysis revealed 7666 statistically significant peaks corresponding to 238 unique genes, ranging in size from 100–416 bp (Table S3). The quality of the chromatin and specificity of the antibody were confirmed by ChIP analysis using the *CDC-42* promoter, previously reported as a putative target of α-synuclein. Additionally, the promoter enrichment was assayed using the *NEDD4* promoter sequence, shown in our analysis to significantly bind α-synuclein (*NEDD4* belongs to the ubiquitin proteasome pathway). Real-time PCR analysis of the immunoprecipitated DNA resulted in a significant enrichment for α-synuclein binding to both the *CDC-42* and *NEDD4* promoter, when compared to the myoglobin exon 2 non-binding control region (2.36- and 1.51-fold enrichment for *CDC-42* and *NEDD4*, respectively).

IPA analysis revealed that the top ten canonical pathways over-represented among the 238 genes targeted by α-synuclein were (Figure S4B): fatty acid biosynthesis (*ACACB*, *OLAH*), mechanisms of viral exit from host cells (*NEDD4*, *PRKCG*, *VPS24*), aldosterone signaling in epithelial cells (*ACCN1*, *NEDD4*, *PIP5K1C*, *PRKCG*), PPAR signaling (*IL1R2*, *INSR*, *MAP4K4*, *PDGFB*), hepatic cholestasis (*ESR1*, *GCGR*, *IL1R2*, *INSR*, *PRKCG*), protein ubiquitination pathway (*AMFR*, *NEDD4*, *PSMD1*, *UBR2*, *USP3*, *USP6*), rac signaling (*ANK1*, *MCF2L*, *PAK3*, *PIP5K1C*), clathrin-mediated endocytosis signaling (*AMPH*, *FGF12*, *FGF13*, *PDGFB*, *PIP5K1C*), glycosphingolipid biosynthesis - ganglioseries (*ELOVL2*, *ST8SIA1*), and ephrin receptor signaling (*EPHA6*, *MAP4K4*, *PAK3*, *PDGFB*, *WIPF1*).

We hypothesized that α-synuclein ChIP peaks/pathways common to predicted target genes/pathways from our miRNA analysis, particularly in the top ten pathways, would highlight important genes and pathways implicated in PD pathology. Glycosphingolipid biosynthesis - ganglioseries together with the protein ubiquitination pathway emerged in both the miRNome and the interacton IPA analyses, with three common genes, *USP6*, *NEDD4*, and *USP3*, in the protein ubiquitination pathway. These three genes are also putative targets of three miRNAs (miR-30b, miR-30c, miR-26a) over-represented in the miRNomic pathway analysis (Table S2).

### Association study

Given that the convergence of the miRNomics and the α-synuclein interacton approaches highlighted multiple genes in the glycosphingolipid biosynthesis and the protein ubiquitination pathways previously been linked to PD [24]–[27], we further investigated the association of the genes in these pathways with risk for PD. Six independent genome-wide association studies investigated the genomic susceptibility to PD [8]–[13], and we performed a joint analysis of the three datasets to which we had access [9], [10], [13]. The meta-dataset in this study includes 1752 PD cases and 1745 controls. We tested the association of 388 SNPs in 20 genes predicted to be targets of 11 out of the 18 differentially expressed miRNAs belonging to the glycosphingolipid biosynthesis - ganglioseries and the protein ubiquitination pathways (Table S4). The number of polymorphisms tested in each gene and the markers with positive association findings are summarized in Table 3.

**Table 3:** Allelic association results for genes in the “miRNA-derived” IPA glycosphingolipid biosynthesis - ganglioseries and protein ubiquitination pathways.

Ingenuity canonical pathway	Gene symbol	miRNA	No. of SNPs tested	SNP	Chr.	Location	p-value
Protein ubiquitination pathway	*UBE4B*	miR-26a	21		1		NS
	*USP37*	miR-30b, miR-30c	58	rs13006838	2	219034545	1.94E-03
				rs13027934		219046825	3.36E-04
				rs10498059		219050149	3.36E-04
				rs7565544		219083908	4.42E-04
				rs13014473		219091935	4.42E-04
				rs6729124		219092735	4.42E-04
				rs13022976		219093770	4.42E-04
				rs6714092		219113849	6.03E-04
				rs6734184		219141633	6.99E-04
	*UBE2F*	miR-30b	5		2		NS
	*UBE2J1*	miR-30b, miR-30c	16		6		NS
	*UBE2H*	miR-335	16		7		NS
	*UBE2D1*	miR-26a	19		10		NS
	*USP15*	miR-26a	21	rs7315790	12	60955680	3.55E-02
				rs10784293		61097595	2.26E-02
	*NEDD4*	miR-30b, miR-30c	27		15		NS
	*USP3*	miR-26a	9	rs10450989	15	61633561	3.97E-02
	*UBE2I*	miR-30b, miR-30c	1		16		NS
	*PSMD7*	miR-30b	2		16		NS
	*UBE2G1*	miR-26a	4		17		NS
	*USP6*	miR-19b	2		17		NS
	*USP32*	miR-19b	7		17		NS
	*NEDD4L*	miR-30b	75	rs878396	18	53962056	1.04E-02
				rs4149589		53966015	1.96E-02
				rs2043265		54206752	2.49E-02
	*USP25*	miR-26a	6		21		NS
Glycosphingolipid biosynthesis - ganglioseries	*DBT*	miR-29b, miR-29c	5		1		NS
	*ST3GAL5*	miR-19b	5		2		NS
	*ST8SIA4*	miR-30b	85	rs2548278	5	100193016	3.39E-02
				rs2059198		100232402	6.15E-03
				rs17160771		100238725	3.61E-02
	*ST8SIA3*	miR-301a	4		18		NS

P-values for significantly associated polymorphisms in the HIHG, CIDR and NINDS meta-dataset are shown.

NS: None significant.

*USP37* (ubiquitin specific peptidase 37) in the protein ubiquitination pathway showed the strongest associations with nine SNPs having p-values between 3.36×10^−4^ and 1.94×10^−3^ (Table 3). All nine SNPs are in high linkage disequilibrium with each other (r^2^>0.9). The most significant SNP rs13027934 (p = 3.36×10^−4^ in the meta-analysis) displayed a consistent association trend in each GWAS dataset: in the Hussman Institute for Human Genomics (HIHG) dataset (OR = 0.57, p = 0.13), in the National Institute of Neurological Disorders and Stroke (NINDS) dataset (OR = 0.46, p = 0.02), in the Center for Inherited Disease Research (CIDR) dataset (OR = 0.55, p = 0.02). Consistent evidence for association was also found for rs2059198 in *ST8SIA4* (ST8 alpha-N-acetyl-neuraminide alpha-2,8-sialyltransferase 4) in the glycosphingolipid biosynthesis pathway (OR = 1.20 and p = 6.15×10^−3^ in the combined GWAS, OR = 1.22 and p = 0.08 in the HIHG dataset, OR = 1.17 and p = 0.36 in the NINDS dataset, and OR = 1.19 and p = 0.05 in the CIDR dataset) (Table 3). The effect sizes of those SNPs are small, which is expected for complex diseases: the median odds ratio for associated SNPs in GWAS is 1.3 [28]. As a result, none of these SNPs would survive the stringent Bonferroni correction for multiple testing given the sample size. The trend for association (in terms of p-values and ORs) in several GWAS datasets and in the meta-analysis, however, supports the possible involvement of these genes in PD etiology.

In addition, there is evidence of association with PD in the meta-analysis for SNPs rs7315790 (p = 3.55×10^−2^) and rs10784293 (p = 2.26×10^−2^) in *USP15* (ubiquitin specific peptidase 15), rs10450989 (p = 3.97×10^−2^) in *USP3* (ubiquitin specific peptidase 3), and rs878396 (p = 1.04×10^−2^), rs4149589 (p = 1.96×10^−2^), and rs2043265 (p = 2.49×10^−2^) in *NEDD4L* (neural precursor cell expressed, developmentally down-regulated 4-like), further supporting the possible involvement of these pathways in PD etiology.

## Discussion

Our data identified a signature of eighteen miRNAs that permits a good separation among PD cases and controls. All of these 18 miRNAs are under-expressed in patients. The slight down-regulation of these miRNAs is predicted to lead to a mild up-regulation of their target genes. A modest over-expression of susceptibility genes may, over time, lead to a late-onset neurodegenerative disease such as PD [29]–[30].

The miR-133b, miR-433 and miR-153 miRNAs previously implicated in PD [3]–[6] were not expressed at detectable levels in the PBMCs samples assayed here, and therefore their role in PD could not be assessed in this study. On the other hand, miR-7 was expressed, but not deregulated in PD. Similar results for miR-133b (not expressed) and miR-433 (not deregulated in PD) were obtained on laser microdissected postmortem dopaminergic neurons in a PD case-control study [29]. Discrepancies in miRNA expression results across studies may result from technical (e.g. sample preparation) as well as biological (e.g. tissue) differences. In our study, we chose to focus on PBMCs not only due to the obvious practical advantages and potential clinical applications of these cells, but also due to important biological considerations. Over 70% of the *substantia nigra* of PD patients is composed of dead or dying cells at the time of diagnosis, and therefore expression studies in SN tissue of PD patients is likely to reveal downstream unspecific processes (such as programmed cell death and survival) rather than pointing to the etiopathogenic mechanisms. Furthermore, brain-enriched miRNAs have limited stability and relatively short half-lives (∼1–3.5 h) in human brain tissues, even with very short post-mortem brain freezing intervals (∼1 h) [31]. There is communication between the brain and immune system through multiple mechanisms that may be reflected in blood cells [32], and PBMCs share more than 80% of the transcriptome with other tissues types including brain [33]. Finally, previous expression profiling studies for neurodegenerative diseases in PBMCs have uncovered important and novel findings [34], and the miRNA expression pattern in normal brain appears to be more similar to PBMCs than to other tissues [35].

The mis-classification of several individuals in HC and PCA analyses could not be correlated to any experimental artefact or error (such as collection date or sample switching), nor to any study participant characteristic (such as age, sex, medication). Even though mis-clustering of individuals is characteristic of high-throughput methods [36], it might have been resolved by the inclusion in the expression study of samples from patients with other parkinsonian syndromes with involvement of the SN such as progressive supranuclear palsy, frontotemporal dementia with parkinsonism [37], and corticobasal degeneration.Consistent with the view that idiopathic PD most likely requires multiple insults in different biological processes before neuronal loss occurs [38], our “miRNA-derived IPA analysis” revealed that several of the top canonical pathways over-represented among our target genes have been previously linked to PD. This is the case for pathways such as the semaphorin signaling in neurons and the DNA methylation and transcriptional repression signaling. Semaphorins are axon guidance proteins playing important roles in the mesencephalic dopamine neuron system development during embryogenesis. A GWAS showed that a SNP in *SEMA5A* was the most significantly associated with PD [8]. However, four subsequent case-control replication studies reported contradictory results [39]–[42]. DNA methylation and transcriptional repression signalling was the third most significantly over-represented pathway. Mechanisms regulating gene expression, whether through DNA methylation or signalling pathways such as checkpoint transduction cascades or transcriptional repression, are very likely associated to PD and regulated by miRNAs. Saijo *et al.* demonstrated that Nurr1/CoREST transrepression pathway attenuates neurotoxic inflammation, protecting against loss of dopaminergic neurons in PD [43]. Also, Zhong *et al.* presented DJ-1 as a potential regulator of protein sumoylation and directly link the loss of DJ-1 expression and transcriptional dysfunction to impaired dopamine synthesis [44].

Genetic and biochemical studies have established a central role for α-synuclein accumulation in the pathogenesis of Parkinson disease. In this study, we identified a large set of putative α-synuclein target (interacting) genes in human H4 neuroglioma cells, which we extensively use as a model for studying the molecular basis of PD [45]–[46], providing the first insight into the interacton of endogenous α-synuclein. Pathway analysis of this interacton suggested several primary targets of α-synuclein, with the glycosphingolipid biosynthesis and the protein ubiquitination pathways being common to the miRNome IPA analysis. Data mining of these pathways in three GWAS studies highlighted the consistent associations of *USP37* and *ST8SIA4* with PD and gave further support to the involvement of glycosphingolipids and the ubiquitin proteasome system in the physiopathology of PD. Furthermore, 3 miRNAs (miR-30b, miR-30c and miR-26a) which are among the most abundant miRNAs in primary human neuronal and glial cells [31] and simultaneously involved in the regulation of α-synuclein interacting genes, emerged as the main modulators of these two pathways in our expression analyses.Glycosphingolipids and their sialic acid-containing derivatives, gangliosides, are important cellular components and abundant in the nervous system. They are known to undergo dramatic changes during brain development, but our knowledge on the mechanisms underlying their quantitative and qualitative changes is still fragmentary [47]. Glycosphingolipids are closely related to the ceramide metabolism that has already been linked to PD through the glucocerebrosidase (*GBA*) gene [26]–[27].

In addition to being the major non-lysosymal system for degrading proteins in the cell, the ubiquitin proteasome system (UPS) regulates function and translocation of proteins, many of which play a role in the determination of cell fate. Protein mediators of apoptosis are regulated by the UPS, via direct or indirect modulation of proteins associated with cell death. Mutations in two PD genes, the E3-ligase *Parkin* and the deubiquitinating enzyme *UCHL1*, may lead to a susceptibility to UPS failure resulting in protein accumulation, Lewy body formation and dopaminergic cell death. Furthermore, dysfunctional α-synuclein and α-synuclein oligomeric species have also been implicated in the impairment of the proteasome system [48], which in turn has been implicated in α-synuclein turnover [15]. Several ubiquitin specific proteases (e.g. *USP24* and *USP40*) have been consistently associated with PD [49]–[52].

To our knowledge, this is the first global miRNAs expression analysis performed in PBMCs in a relatively large cohort of PD patients and controls. Taken together, this work suggests that a small subset of miRNAs may act as regulators of cellular mechanisms leading to PD. Some of the pathways highlighted here are already known to be involved in PD pathogenesis, but others constitute new avenues for future research.

## Materials and Methods

### Ethics Statement

The Lisbon University Hospital's ethical committee approved the study, and all participants or their legal representatives signed an informed consent.

### Patient and control samples

Study participants were recruited at the movement disorders outpatient clinic of the Lisbon University Hospital over a period of eight months. Patients were evaluated by neurologists with expertise in PD. Diagnosis of Parkinson's disease was based on the UK Parkinson's Disease Society Brain Bank's criteria [53]. All recruited patients had more than two years of disease duration, therefore significantly reducing the probability of including atypical or secondary causes of parkinsonism [53]. Controls demonstrated no signs of parkinsonism and had no family history of PD. Patients' clinical assessment included the modified Hoehn and Yahr staging [54], the Schwab and England Activities of Daily Living Scale [55], and the Unified Parkinson's Disease Rating Scale (UPDRS) [56]. All participants were Portuguese Caucasians.

### Sample collection and RNA isolation

Approximately 16 ml of whole blood was collected per participant by venipuncture into two BD Vacutainer CPT glass tubes (Becton-Dickinson). Within two hours of blood collection, PBMCs were isolated from the CPT tubes by centrifugation (30 minutes, 1639 *g*, room temperature), washed twice with PBS (15 minutes, 353 *g*, 4°C) and conserved on RNA later (Qiagen). Total RNA containing small RNAs species was extracted from PBMCs using the miRNeasy Mini kit (Qiagen) according to the manufacturer's instructions, aliquoted and stored at −80°C. Since high-quality RNA is an absolute prerequisite to obtain reliable and reproducible microarray data, all samples passed an extensive quality control (e.g. OD_260_/OD_280_ between 1.8 and 2.1 in the Nanodrop ND1000 and RNA Integrity Number>7 in the Agilent 2100 Bioanalyser).

### miRNA profiling

RNA samples were sent to Exiqon (Denmark) for miRNA profiling. Briefly, 1 µg of total RNA from samples meeting the quality standards described above were labeled with the miRCURY™ Hy3™/Hy5™ power labeling kit and hybridized on the miRCURY™ locked nucleic acid (LNA) array (version 10.0) using a Tecan HS4800 hybridization station. Each sample was co-hybridized with a Hy5-labeled common reference pool composed of equal RNA concentration of the 39 samples (the 32 under study and 7 additional samples, later excluded from differential expression analysis due to array quality control problems). Arrays were scanned with an Agilent G2565BA Microarray Scanner System and stored in an ozone-free environment in order to prevent potential bleaching of the fluorescent dyes. Image analysis was carried out using the ImaGene 7.0 software (BioDiscovery) and the resulting ImaGene output files were analyzed using the Limma package [16] implemented in the R freeware (http://cran.r-project.org/). Microarray data submission for human arrays is MIAME compliant. Raw data from the microarray study have been deposited in the Gene Expression Omnibus (GEO) database with the accession number GSE16658. Background correction was performed using the normexp method (with offset = 10). Intensities of good quality spots were then subjected to within-array (loess method) normalization. Intensities lower than 1.5 times the median signal intensity of the respective slide and label were discarded. The “calling” of a miRNA on a particular array fails if two or more of the four replicated capture probes for this miRNA are flagged as bad quality spots by the image analysis software. Differential miRNA expression between controls and PD patients was assessed using linear regression analysis and empirical Bayes methods implemented in the Limma package.

Hierarchical clustering and principal component analysis of the samples were performed with the Partek Genomics Suite software v.6.5 using the log_2_(sample/reference) ratios of the 18 differentially expressed miRNAs as input. HC was performed using the Euclidean distance function and the average linkage clustering methods. PCA analysis was set to run using the following options: covariance dispersion matrix (used when the variables are measured in the same units and have similar variances) and normalized eigenvector scaling (orthogonal eigenvectors and scaled to unit).

### Chromatin immunoprecipitation followed by sequencing analysis

Human H4 neuroglioma cells (HTB-148-ATCC, Manassas) were maintained in OPTI-MEM (Life Technologies) supplemented with 10% fetal bovine serum and maintained at 5% CO_2_, 37°C to confluence. The cells were cross-linked in 1% formaldehyde for 10 minutes at room temperature and pelleted by low-speed centrifugation (1000 *g*). The cells were then lysed in lysis buffer (1.0% SDS, 10 mM EDTA, 50 mM Tris-HCl pH 8.1) containing Protease Inhibitor Cocktail (Roche). Chromatin was sheared to an average DNA fragment size of 100–500 bp by sonication using the Diagenode Bioruptor sonicator (2×15 minutes of 30-second bursts at high output power with 30-second rest intervals between pulses) and fragment sizes were checked by agarose gel electrophoresis and with the Agilent Bioanalyzer DNA 1000 kit. 100 µl of chromatin was aliquoted as total input (Input) and immunoprecipitated (IP) for later steps. Chromatin was precleared with 45 µl Protein G-Agarose solution (Upstate), followed by the immunoprecipitation using 2.5 µg of α-synuclein antibody (Ref. No. 610786, BD Biosciences) overnight at 4°C with slow rotation. Subsequently, the beads were washed using Buffer 1 (0.1% SDS, 1% Triton X-100, 2 mM EDTA, 20 mM Tris-HCl pH 8.1, 100 mM NaCl), Buffer 2 (Buffer 1 with 500 mM NaCl), Buffer 3 (0.25 M LiCl, 1% NP-40, 1% C_24_H_39_NaO_4_, 1 mM EDTA, 10 mM Tris-HCl), and 2× TE Buffer. Chromatin was eluted by vortexing and rotation at room temperature in 1% SDS and 0.1 M NaHCO_3_. To reverse the crosslinks, precipitated chromatin was incubated at 65°C overnight by adding NaCl (to 0.2 M) and then phenol-extracted. The DNA was purified using the QIAquick PCR Purification Kit (Qiagen). The total amount of DNA was measured using PicoGreen (Invitrogen) and a NanoDrop Spectrophotometer. Immunoprecipitated DNA and total input DNA were blunt ended and adapters were ligated to the ends, according to the library preparation protocol from Illumina. The DNA fragments with 200±25 bp in length were then selected for the construction of the ChIP-seq DNA library. The quality of the libraries was checked using the Agilent Bioanalyzer HS DNA Kit, showing correct size and concentration of the samples. Following the size selection, all the resulting immunoprecipitated DNA fragments were amplified and sequenced simultaneously using the Illumina Genome Analyzer II.

Using the ELAND software (Illumina), 9.694.993 reads were aligned to the repeat masked NCBI 36/hg18 build of the human genome, allowing up to two mismatches. The Partek Genomics Suite (Partek) ChIP-seq analysis workflow was utilized to identify α-synuclein enriched regions. The software uses a sliding window approach to identify regions of higher read depth, referred to as peaks, relative to a background distribution. We used a 100 bp window and the zero-truncated binomial model with a peak cut-off p-value of 0.001.

### Validation of ChIP-seq results by ChIP real-time PCR

The original IP and Input DNA aliquots from the microarray experiment were used to assess the quality of the ChIP. α-synuclein occupancy was detected by qPCR at the *CDC-42* and *NEDD4* promoters. Primers were designed with the PRIMER3 program (http://frodo.wi.mit.edu/cgi-bin/primer3/primer3-www.cgi). The primer sequences were as follows: *CDC-42* forward GATGCACCACTGTTCTCCAG, reverse AGACACCTCTCCTCCTTCCAC; *NEDD4* forward GTAACGAGCCCTTCCTGTGA, reverse TCAACTACCCGACCGACTTC; myoglobin exon 2 forward AAGTTTGACAAGTTCAAGCACCTG, reverse TGGCACCATGCTTCTTTAAGTC. The qPCR was performed on a Roche LightCycler in triplicate. The quality of the ChIP was calculated as fold enrichment over myoglobin exon 2 non-binding control region.

### Pathway analyses

Predicted target genes or the α-synuclein interacting genes were uploaded into the commercially available IPA software v.7.6 for biological function and pathway analysis. IPA is built upon a very large manually-curated and up-to-date database of genes, proteins, functions, interactions/networks, and pathways. The IPA “Core Analysis” was performed using the Ingenuity Knowledge Base (IPAKB, Genes only) as the reference set, using direct and indirect relationships for network analysis, and data from mammal species, and all tissues, cell lines and data sources. The significance (p-values) of the association between a dataset and a canonical pathway was determined by comparing the number of genes in a dataset that participate in a given pathway to the total number of occurrences of these genes in all pathway annotations that are stored in the IPAKB. A Fisher's exact test was used to calculate the p-value to determine the probability that the association between the genes in the dataset and the canonical pathway is explained only by chance. The level of statistical significance was set to p<0.05. Each pathway analysis generated the top canonical pathways with a statistical significance (Figures S4A and S4B).

### Whole-genome association data mining

A joint association analysis was performed on three PD GWAS datasets: The HIHG at the University of Miami [13], the NINDS [9], and the joint dataset from the Progeni/GenePD studies that was genotyped at the CIDR [10]. This meta-dataset has a combined sample size of 1752 cases and 1745 controls. Details regarding the characteristics of the study participants and the markers analyzed in each dataset are described in detail in the original reports [9], [10], [13]. Briefly, genotype and phenotype data from the NINDS and Progeni/GenePD studies were downloaded from dbGAP (http://www.ncbi.nlm.nih.gov/sites/entrez?db=gap). HIHG genotype data were generated using the Illumina Infinium 610-quad BeadChip. Imputation of SNP genotypes from the three GWAS dataset was performed using the software package Impute [57]. Samples with a genotyping efficiency <0.98 and SNPs with a genotyping call rate <0.98 were removed from the analysis. In addition, SNPs with a minor allele frequency <0.01 or a Hardy-Weinberg equilibrium p-value<10^−7^ (in controls) were excluded. Population stratification was evaluated using Eigenstrat [58]. Cochran-Armitage trend tests were used to assess allelic association at each SNP using PLINK [59].

## Supporting Information

Figure S1 **Validation of microarray expression results by qRT-PCR of five miRNAs.** Five miRNAs showing distinctive expression patterns between PD and control samples were selected for global validation of the microarray results by qRT-PCR on the same dataset of 19 PD cases and 13 controls. We selected miRNAs (miR-126*, miR-32 and miR-101) that were under-expressed (fold-change <−1), and miRNAs (miR-15b and miR-550) that were over-expressed (fold-change>1) in the microarrays, both above (miR-126*) and below (miR-32, miR-101, miR-15b, and miR-550) our threshold for significance (B-statistic≥1). Normalization to endogenous expression levels of control genes is currently the most accurate method to correct for potential RNA input or RT efficiency biases. The NormFinder algorithm [61] was used to assess the variance in expression levels in our miRNAs and to select the best candidate endogenous controls (three small nuclear/nucleolar RNAs RNU66, RNU6B and Z30, and miRNA miR-103) for qPCR normalization. Quantification of selected miRNAs and endogenous controls by TaqMan® Real-Time PCR was carried out as described by the manufacturer (Applied Biosystems [ABI]). Briefly, 50 ng of template RNA was reverse transcribed using the TaqMan® MicroRNA Reverse Transcription Kit and stem-loop miRNA-specific primers from the ABI assays (TaqMan® MiRNA assays miR-126*, miR-32, miR-101, miR-15b, miR-550, miR-103, RNU66, RNU6B and Z30). 1.33 µl of each RT product was PCR-amplified in triplicate in 20 µl PCR reactions (95°C for 10 minutes, followed by 50 cycles of 95°C for 15 seconds and 60°C for 1 minute) in 384-well plates on the ABI 7900HT Fast Real-Time PCR System (ABI). For each assay, a paired no-template control (NTC) and a common reference pool (equal quantity of RNA from the 19 cases and 13 controls) reactions were performed. Based on the qRT-PCR results, miR-103 was selected by NormFinder as the best endogenous control (stability value of 0.223) and was further used as the reference miRNA to normalize expression levels. Expression values were calculated using the ΔΔC_T_ method: 2^−(ΔC^_T_^[sample]−ΔC^_T_^[pool])^ with ΔC_T_ = C_T_(selected miRNA)−C_T_(miR-103) [62]. The PD/control expression ratio (expected value between 0 and +∞) is calculated by dividing the average ΔΔC_T_ of cases by the average ΔΔC_T_ of controls. Ratio values between 0 and 1 indicate that a miRNA is down-regulated in PD patients, and values greater than 1 mean that it is up-regulated in PD patients. Ratio values can be converted to the more intuitive fold-change (FC) values by taking the negative inverse (−1/ratio) for ratios between 0 and 1 (e.g. a ratio of 0.5 corresponds to a −2 FC, which means that the miRNA is 2-fold down-regulated in cases). FC values are equal to ratios for ratios greater than one. The expected values of FC are ]− ∞;−1] and [1;+∞[. A two-tailed t-test with unequal variance was used to assess significant differences in qRT-PCR among cases and controls. The table below the graph indicates the fold-changes and B-statistics obtained for the selected miRNAs in the microarray experiment, as well as the fold-changes and p-values obtained in the qRT-PCR experiment.
(PDF)

Figure S2 **Two-dimensional principal component analysis (PCA) plot of the 32 individuals.** PCA analysis was based on the expression levels of the eighteen differentially expressed miRNAs. Control and patient samples are shown in black and red, respectively. Males are represented by squares and females by circles, and the individual's ID (Table 1) is indicated inside the symbol.
(PDF)

Figure S3 **Sources of variation in the miRNomics expression data.** Analysis of variance (3-way ANOVA) was used to evaluate the impact of several potential sources of variation (age, gender and affection status) in our expression data.
(PDF)

Figure S4 **Top ten canonical pathways over-represented among the predicted target genes of 11 out of the 18 differentially expressed miRNAs (A) and among the 238 genes targeted by α-synuclein (B).** These analyses were carried out using the Ingenuity Pathway Analysis software and each plot displays the pathways ranked by significance level on the y-axis. The x-axis on the top is for the negative logarithm of the p-value (orange dots connected by a line). The significance (p-values) of the association between a dataset and a canonical pathway was determined by comparing the number of genes in a dataset that participate in a given pathway to the total number of occurrences of these genes in all pathway annotations that are stored in the Ingenuity Knowledge Base. The p-value is calculated using the right-tailed Fisher exact test to determine the probability that the association between the genes in the dataset and the canonical pathway is explained only by chance. The number of genes in our dataset in a given pathway and the total number of genes associated with that pathway in the IPA's database are shown to the right of each pathway and are used to calculate the percentage displayed in the grey bars (the x-axis on the bottom is for the grey bars). The percentage and the significance are measures of the amount and confidence, respectively, of association of a given canonical pathway with the data.
(PDF)

Table S1 959 target genes (662 unique genes) predicted by miRecords for 11 differentially expressed miRNAs. Only genes that are predicted to be targets by at least six softwares are shown.
(XLS)

Table S2 Top ten canonical pathways in the Ingenuity Pathway Analysis of the 662 genes predicted to be targets of 11 differentially expressed miRNAs. The target genes and their respective miRNA in each pathway are indicated. The ratio corresponds to the number of genes in a pathway that were in our target gene list divided by the total number of genes in that pathway. The p-value is calculated using the right-tailed Fisher exact test and takes into account the number of genes in our dataset in a given pathway and the total number of genes associated with that function in IPA's database. The ratio and the significance are measures of the amount and confidence, respectively, of association of a given canonical pathway with the data.
(XLS)

Table S3 ChIP-seq target genes containing the α-synuclein enriched regions overlapping with the transcribed sequences.
(XLS)

Table S4 Allelic association results for SNPs in the glycosphingolipid biosynthesis - ganglioseries and the protein ubiquitination pathway target genes.
(XLS)
