# Supplementary material for: Convergence of miRNA Expression Profiling, α-Synuclein Interacton and GWAS in Parkinson's Disease
Source: PLoS One. 2011 Oct 7;6(10):e25443. doi: 10.1371/journal.pone.0025443 (PMC3189215; doi:10.1371/journal.pone.0025443)
Supplement: Figure S1 — Validation of microarray expression results by qRT-PCR of five miRNAs. Five miRNAs showing distinctive expression patterns between PD and control samples were selected for global validation of the microarray results by qRT-PCR on the same dataset of 19 PD cases and 13 controls. We selected miRNAs (miR-126*, miR-32 and miR-101) that were under-expressed (fold-change <−1), and miRNAs (miR-15b and miR-550) that were over-expressed (fold-change>1) in the microarrays, both above (miR-126*) and below (miR-32, miR-101, miR-15b, and miR-550) our threshold for significance (B-statistic≥1). Normalization to endogenous expression levels of control genes is currently the most accurate method to correct for potential RNA input or RT efficiency biases. The NormFinder algorithm [61] was used to assess the variance in expression levels in our miRNAs and to select the best candidate endogenous controls (three small nuclear/nucleolar RNAs RNU66, RNU6B and Z30, and miRNA miR-103) for qPCR normalization. Quantification of selected miRNAs and endogenous controls by TaqMan® Real-Time PCR was carried out as described by the manufacturer (Applied Biosystems [ABI]). Briefly, 50 ng of template RNA was reverse transcribed using the TaqMan® MicroRNA Reverse Transcription Kit and stem-loop miRNA-specific primers from the ABI assays (TaqMan® MiRNA assays miR-126*, miR-32, miR-101, miR-15b, miR-550, miR-103, RNU66, RNU6B and Z30). 1.33 µl of each RT product was PCR-amplified in triplicate in 20 µl PCR reactions (95°C for 10 minutes, followed by 50 cycles of 95°C for 15 seconds and 60°C for 1 minute) in 384-well plates on the ABI 7900HT Fast Real-Time PCR System (ABI). For each assay, a paired no-template control (NTC) and a common reference pool (equal quantity of RNA from the 19 cases and 13 controls) reactions were performed. Based on the qRT-PCR results, miR-103 was selected by NormFinder as the best endogenous control (stability value of 0.223) and was further used as the reference miRNA to n [file pone.0025443.s001.pdf]

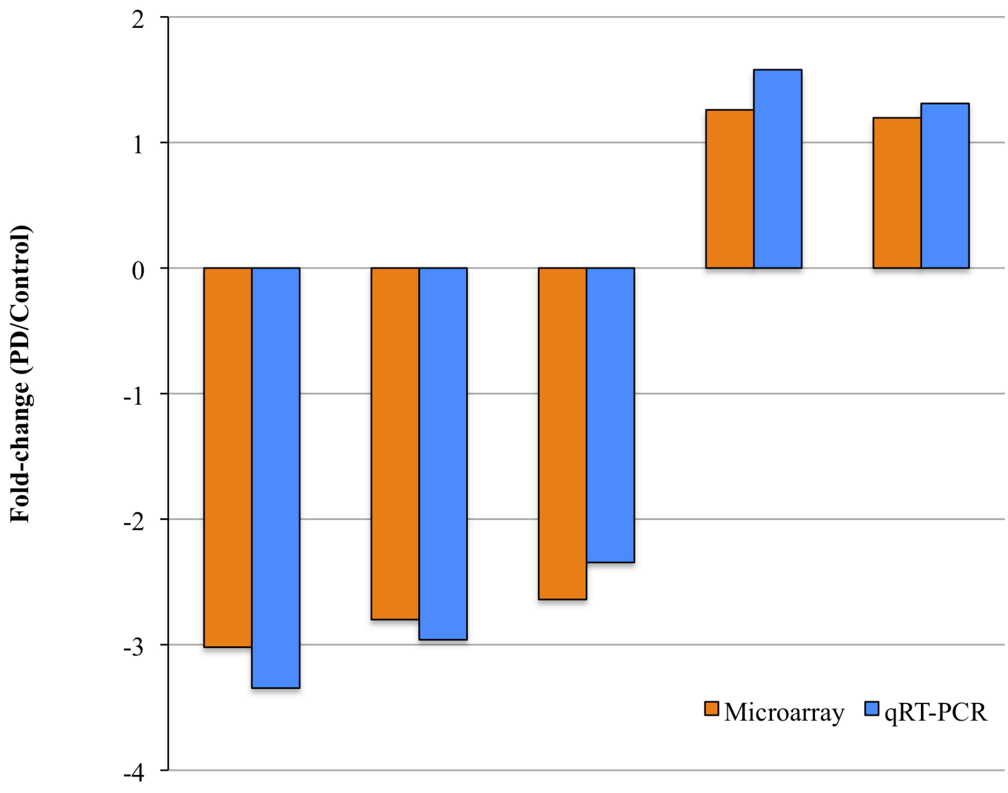

		miR-126*	miR-32	miR-101	miR-15b	miR-550
Microarray	Fold-change	-3.02	-2.80	-2.64	1.26	1.20
	B-statistic	2.68	0.63	0.56	-3.17	-4.49
qRT-PCR	Fold-change	-3.35	-2.96	-2.35	1.58	1.31
	p-value	2.00E-03	9.00E-03	1.63E-02	1.58E-02	1.53E-01

Figure S1
