# Supplementary figures and images for: Convergence of miRNA Expression Profiling, α-Synuclein Interacton and GWAS in Parkinson's Disease
Source: PLoS One. 2011 Oct 7;6(10):e25443. doi: 10.1371/journal.pone.0025443 (PMC3189215; doi:10.1371/journal.pone.0025443)

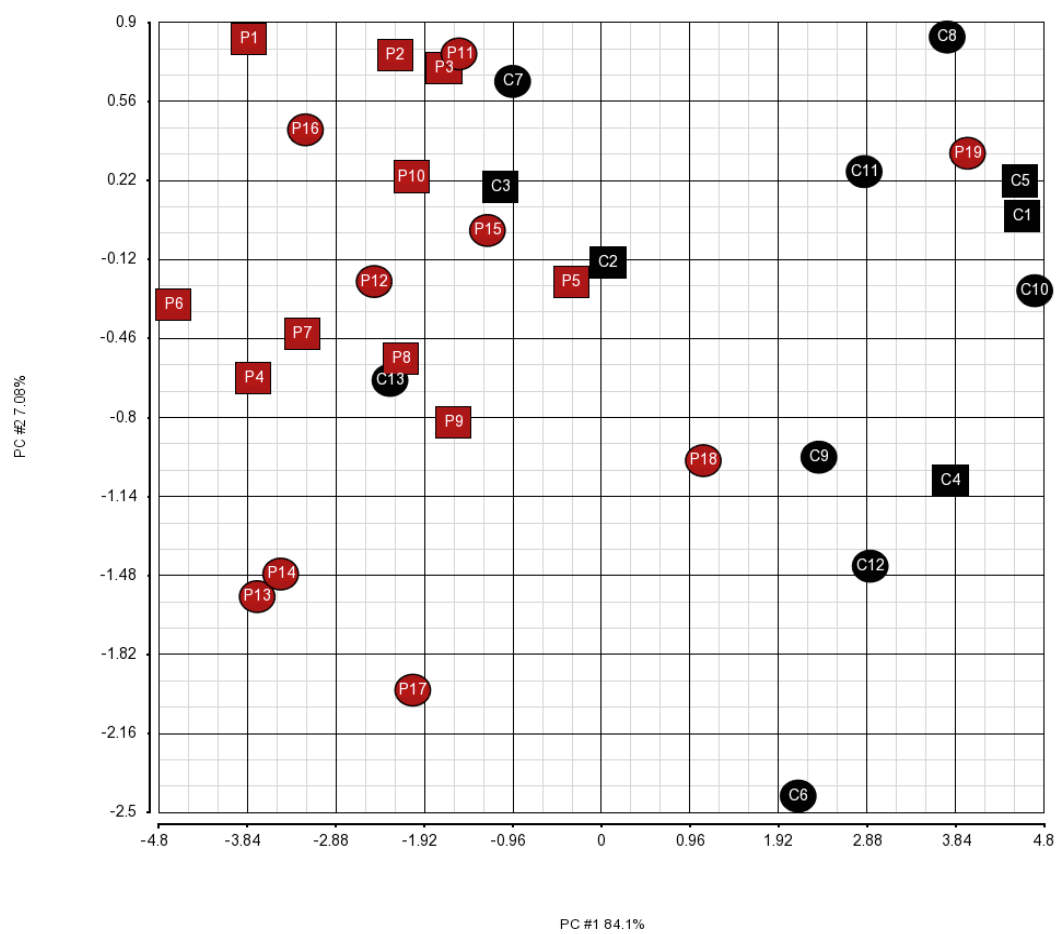

**Figure S2**

Supplement: Figure S2 — Two-dimensional principal component analysis (PCA) plot of the 32 individuals. PCA analysis was based on the expression levels of the eighteen differentially expressed miRNAs. Control and patient samples are shown in black and red, respectively. Males are represented by squares and females by circles, and the individual's ID (Table 1) is indicated inside the symbol. (PDF) [file pone.0025443.s002.pdf]

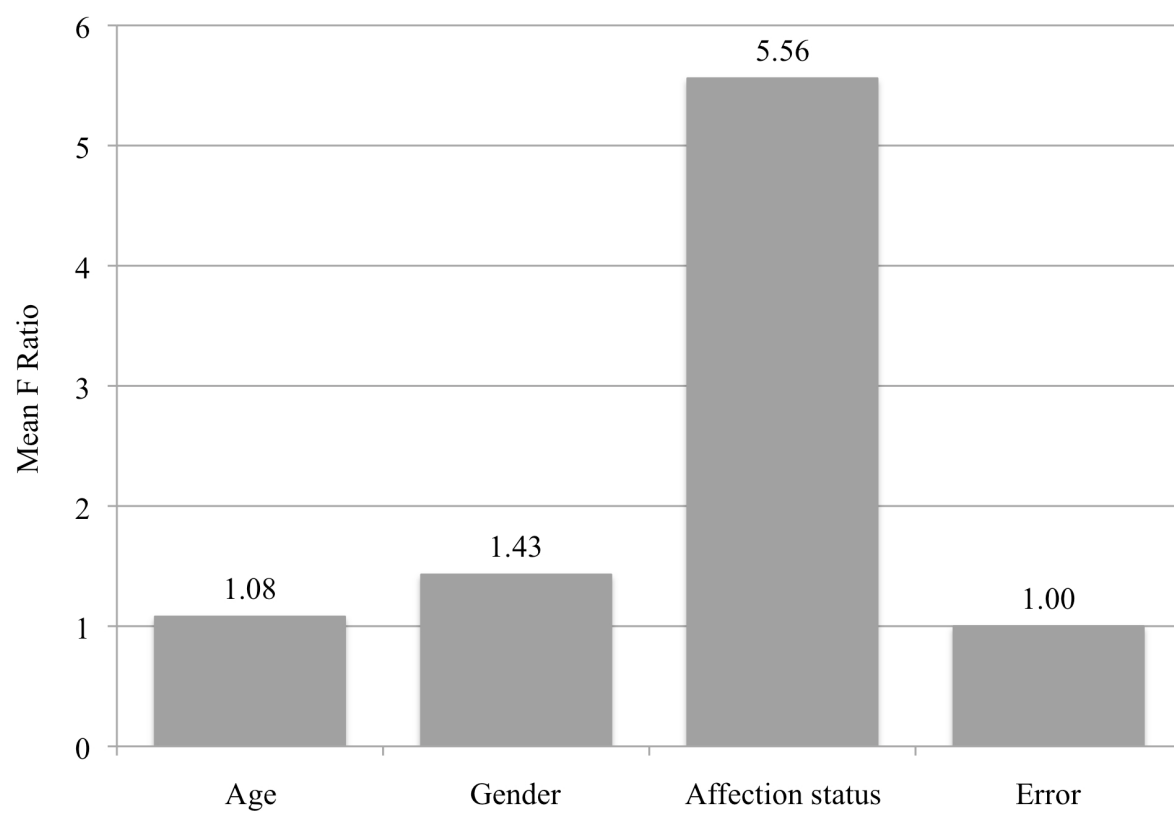

**Figure S3**

Supplement: Figure S3 — Sources of variation in the miRNomics expression data. Analysis of variance (3-way ANOVA) was used to evaluate the impact of several potential sources of variation (age, gender and affection status) in our expression data. (PDF) [file pone.0025443.s003.pdf]

A

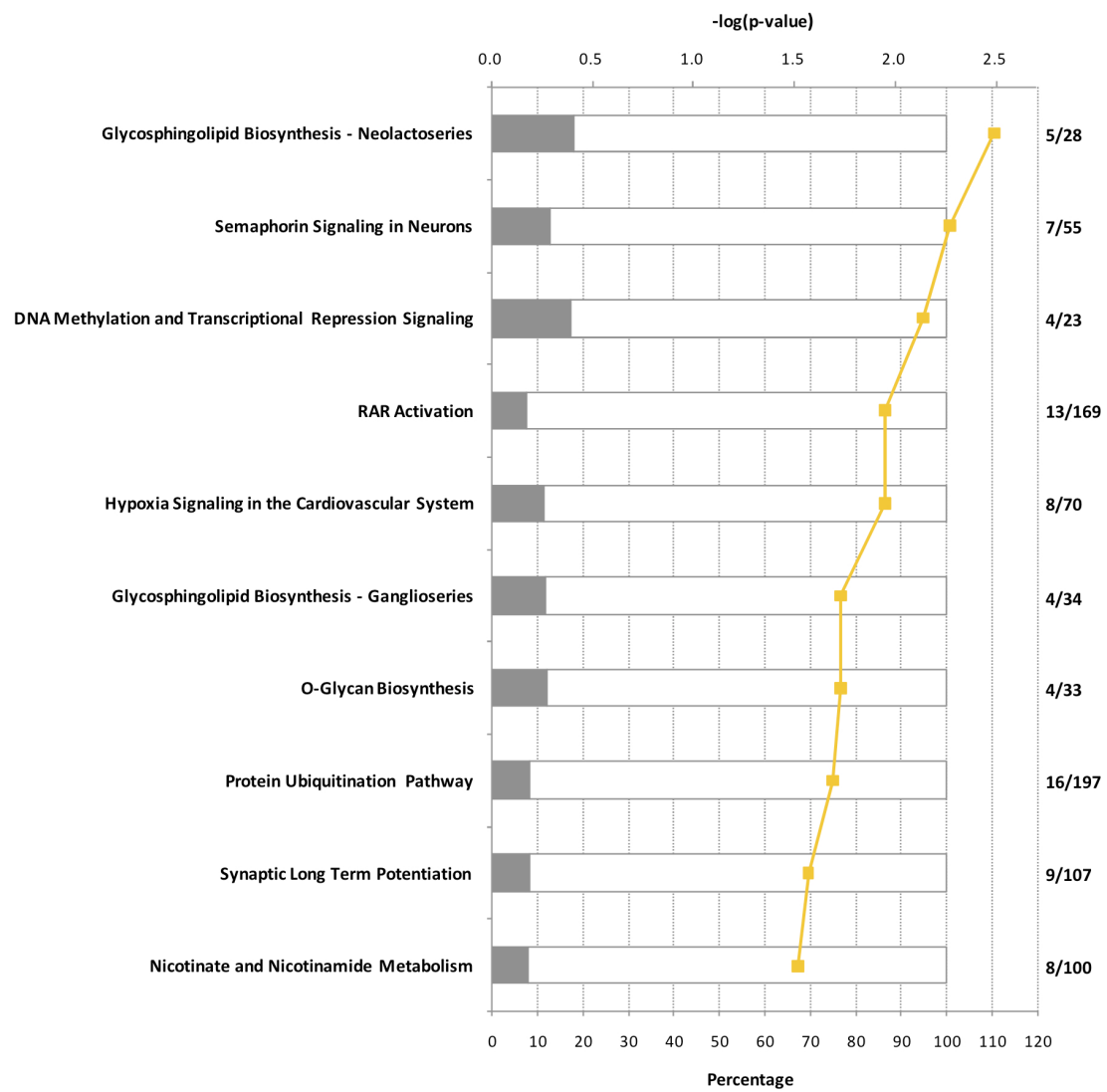

B

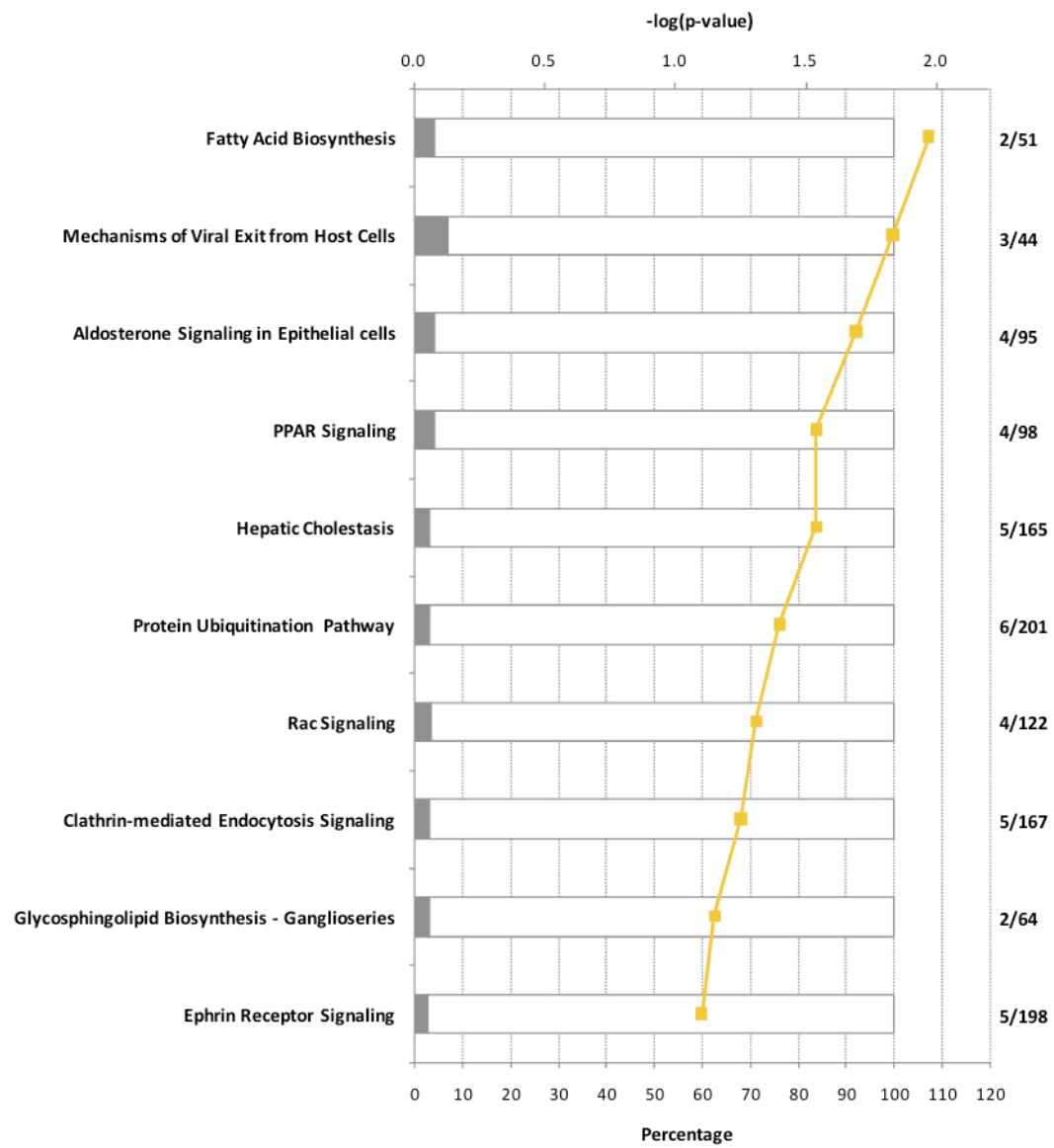

Figure S4

Supplement: Figure S4 — Top ten canonical pathways over-represented among the predicted target genes of 11 out of the 18 differentially expressed miRNAs (A) and among the 238 genes targeted by α-synuclein (B). These analyses were carried out using the Ingenuity Pathway Analysis software and each plot displays the pathways ranked by significance level on the y-axis. The x-axis on the top is for the negative logarithm of the p-value (orange dots connected by a line). The significance (p-values) of the association between a dataset and a canonical pathway was determined by comparing the number of genes in a dataset that participate in a given pathway to the total number of occurrences of these genes in all pathway annotations that are stored in the Ingenuity Knowledge Base. The p-value is calculated using the right-tailed Fisher exact test to determine the probability that the association between the genes in the dataset and the canonical pathway is explained only by chance. The number of genes in our dataset in a given pathway and the total number of genes associated with that pathway in the IPA's database are shown to the right of each pathway and are used to calculate the percentage displayed in the grey bars (the x-axis on the bottom is for the grey bars). The percentage and the significance are measures of the amount and confidence, respectively, of association of a given canonical pathway with the data. (PDF) [file pone.0025443.s004.pdf]
